# Supplementary material for: Hot-carrier tunable abnormal nonlinear absorption conversion in quasi-2D perovskite
Source: Nat Commun. 2022 Nov 14;13:6935. doi: 10.1038/s41467-022-34705-8 (PMC9663601; doi:10.1038/s41467-022-34705-8)
Supplement: Supplementary file 1 — Supplementary information [file 41467_2022_34705_MOESM1_ESM.pdf]

**Supplementary information**

# Hot-carrier tunable abnormal nonlinear absorption conversion in quasi-2D perovskite

Gang Wang[1], Tanghao Liu[1, 2], Bingzhe Wang[1], Hao Gu[1], Qi Wei[3], Zhipeng Zhang[1], Jun He[4]*, Mingjie Li[3, 5]*, Guichuan Xing[1]*

[1]Joint Key Laboratory of the Ministry of Education, Institute of Applied Physics and Materials Engineering, University of Macau, Avenida da Universidade, Taipa, Macao SAR 999078, China

Email: gcxing@um.edu.mo

[2]Department of Physics, Hong Kong Baptist University, 224 Waterloo Road, Kowloon, Hong Kong SAR, 999077, China

[3]Department of Applied Physics, The Hong Kong Polytechnic University, Hong Kong, China

E-mail: ming-jie.li@polyu.edu.hk

[4]Hunan Key Laboratory of Super Microstructure and Ultrafast Process, School of Physics and Electronic, Central South University, 932 South Lushan Road, Changsha 410083, China

E-mail: junhe@csu.edu.cn

[5]Photonics Research Institute, The Hong Kong Polytechnic University, Hung Hom, Kowloon, Hong Kong

# Catalogue

Supplementary Note 1: Nonlinear absorption conversion mechanism.
Supplementary Table 1. Summary of the calculated RSA-SA conversion threshold of reported organic molecules under 100 fs laser pulse excitation.
Supplementary Note 2: Standard nonlinear absorption and saturable absorption fitting model.
Supplementary Figure 1. Z-scan of $(PEA)_2FAPb_2I_7$ (n=2) quasi-2D perovskite film.
Supplementary Figure 2. Z-scan of $FA_{0.9}MA_{0.1}PbI_3$ 3D perovskite film.
Supplementary Table 2. Summary of the NLO parameters of $(PEA)_2FAPb_2I_7$ quasi-2D perovskite film.
Supplementary Note 3: Initial photon-injecting carrier density calculation.
Supplementary Figure 3. Wavelength-dependent TA spectra.
Supplementary Figure 4. Power-dependent TA spectra.
Supplementary Figure 5. Wavelength-dependent absorption singularity.
Supplementary Figure 6. Power-dependent absorption singularity.
Supplementary Figure 7. Ultrafast thermalization and hot carrier cooling kinetics.
Supplementary Table 3. Summary of the fitting parameters using NLA conversion model.
Supplementary Note 4: Linear and transient absorption spectrum of semiconductor.
Supplementary Figure 8. Linear absorption spectra fitting.
Supplementary Figure 9. TA spectra fitting.
Supplementary Figure 10. TA kinetics at band-edge.
Supplementary Note 5: NLA simulation on the basic of non-thermalized and hot carrier model
Supplementary Table 4. The parameters using in NLA simulation.
Supplementary Figure 11. NLA simulation with different parameters.
Supplementary Figure 12. Linear absorption spectra of different 2D perovskite materials.
Supplementary Figure 13. Z-scan of $(PEA)_2FAPb_2Br_7$ (n=2) quasi-2D perovskite film.
Supplementary Figure 14. TA spectrum of $(PEA)_2FAPb_2Br_7$ (n=2) quasi-2D perovskite film.
Supplementary Figure 15. Z-scan of $(PEA)_2PbI_4$ (n=1) 2D perovskite film.
Supplementary Figure 16. TA spectrum of $(PEA)_2PbI_4$ (n=1) 2D perovskite film.
Supplementary Figure 17. TA spectrum of the high-dimensional doping phase.
Supplementary Figure 18. Autocorrelation signal (a) and spectrum (b) of the used femtosecond laser pulse in experiment.
Supplementary Figure 19. The schematic diagram of the Z-scan system.

## List of Acronyms

| Acronyms | Definition |
|---|---|
| AFM | Atomic Force Microscope |
| BGR | Band-Gap Renormalization |
| CB | Conduction Band |
| HC | Hot Carrier |
| ESA | Excited State Absorption |
| MPA | Multiphoton Absorption |
| NLA | Nonlinear Absorption |
| NLO | Nonlinear Optics/Optical |
| NTC | Non-Thermalized Carrier |
| PIB | Photon-Induced Bleaching |
| PIA | Photon-Induced Absorption |
| QW | Quantum-Well |
| RSA | Reverse Saturable Absorption |
| SA | Saturable Absorption |
| SEM | Scanning Electron Microscope |
| 2D | Two-Dimensional |
| 3D | Three-Dimensional |
| TA | Transient Absorption |
| 2PA | Two-Photon Absorption |
| VB | Valence Band |
| XRD | X-ray Diffraction |

**Supplementary Note 1: Nonlinear absorption conversion mechanism.**

Here, we consider the overlapping between standard saturable absorption (SA) and reverse-saturable absorption (RSA) stemming from the lowest third-order nonlinear susceptibility. When the RSA and SA coexist, the total absorption coefficient $\alpha$ can be expressed as:[1]

$$\alpha = \frac{\alpha_0}{1 + {}^{I}/_{I_s}} + \beta I \quad (1)$$

Where $\alpha_0$ is the linear absorption coefficient, $I$ is the incident laser intensity, $\beta$ is the RSA coefficient, $I_s$ is the saturable absorption intensity. Hence, the absorption difference $\Delta\alpha$ can be calculated by:

$$\Delta\alpha = \alpha - \alpha_0 = \beta I - \frac{{}^{\alpha_0}/_{I_s} \cdot I}{1 + {}^{I}/_{I_s}} \quad (2)$$

At low power condition, above equation is simplified as $\Delta\alpha = (\beta - \alpha_0/I_s)I$ . If $\beta < \alpha_0/I_s$, $\Delta\alpha$ is less than 0 under low excitation intensity, which indicates SA. With the pump power increasing, the SA terms reach saturation, but the RSA is not be limited. Hence, the RSA will surpass the SA finally to result in a conversion from SA to RSA

(Figure 1a in main text). However, if $\beta > \alpha_0/I_s$, the $\Delta\alpha$ will maintain larger than 0 regardless at low or high excitation density and we can only observe RSA response. Thus, the combination of SA and RSA only will lead to the conversion from SA to RSA with laser fluence rising.

Additionally, for organic molecules, the RSA is ascribed to strong excited state absorption (ESA) as illustrated in Fig. 1b in main text. the NLA evolution can be described by a set of rate equations for a multi-energy-level:[2]

$$\begin{cases} \frac{dN_0}{dt} = -\sigma_{10}N_0\frac{I}{\hbar\omega} + \frac{N_1}{\tau_{01}} \\ \frac{dN_1}{dt} = \sigma_{10}N_0\frac{I}{\hbar\omega} - \sigma_{21}N_1\frac{I}{\hbar\omega} - \frac{N_1}{\tau_{01}} + \frac{N_2}{\tau_{12}} \\ \frac{dN_2}{dt} = \sigma_{21}N_1\frac{I}{\hbar\omega} - \frac{N_2}{\tau_{12}} \\ N_0 + N_1 + N_2 = N \\ \frac{dI}{dz} = -(\sigma_{10}N_0 + \sigma_{21}N_1 + \sigma_{32}N_2)I \end{cases} \quad (3)$$

where $N$ is the total population, $N_0$, $N_1$, $N_2$ and $N_3$ are populations in energy level $S_0$, $S_1$, $S_2$ and higher energy level. $\tau_{01}$ and $\tau_{12}$ are the lifetimes of energy level $S_1$ and $S_2$, respectively. $I$ is the incident intensity and z is the propagation direction. Here, the population $N_3$ is assumed to be zero owing to the rapid relaxation of the higher energy level. According to this model, the NLA response is determined by the absorption cross-section $\sigma_{10}$, $\sigma_{21}$ and $\sigma_{32}$. Only when the absorption cross-section satisfies $\sigma_{10} < \sigma_{21} > \sigma_{32}$, the RSA-SA conversion will occur at high pump fluence. Utilizing this method, we calculate the NLA conversion threshold $I_C$ for some typical organic molecules which have been reported to suggest RSA-SA switching, under 100 fs laser pulse excitation. The results are summarized in Supplementary Table 1, where the organic molecules suggest a high value of $I_C$ up to a few hundred GW $cm^{-2}$.

**Supplementary Table 1. Summary of the calculated RSA-SA conversion threshold of reported organic molecules under 100 fs laser pulse excitation.**

| Organic molecules | $\sigma_{10}$ ($10^{17}$ $cm^2$) | $\sigma_{21}$ ($10^{16}$ $cm^2$) | $\sigma_{32}$ ($10^{18}$ $cm^2$) | $\tau_{01}$ (ps) | $\tau_{12}$ (ps) | Conversion threshold $I_C$* (GW $cm^{-2}$) | Ref. |
|---|---|---|---|---|---|---|---|
| HITCI | 1.67 | 4.84 | 1.6 | 1500 | 3 | 49 | 3 |
| HITCI | 1.67 | 4.84 | 0 | 1500 | 10 | 45.5 | 4 |
| CuP | 0.2 | 0.35 | 0 | 100 | - | 492 | 5 |
| (TXP)Cd | 2.45 | 1.0 | 3.0 | 100 | 4.8 | 77 | |
| [(TXP)Cd]Cl | 1.3 | 1.0 | 3.0 | 100 | 4.7 | 121 | 6 |
| Polymethine dyes | 0.7~3.2 | 0.55~60 | - | 30~ 800 | - | 45~201 | 7 |

*Calculated with Eq. 3 in **Supplementary Note 1**.

**Supplementary Note 2: Standard nonlinear absorption and saturable absorption fitting model.**

For a typical reverse saturable absorption (RSA), the total absorption coefficient can be expressed as:

$$\alpha = \alpha_0 + \beta I \tag{4}$$

Where $\alpha_0$ is the linear absorption coefficient and $\beta$ is the NLA coefficient, $I$ is the incident laser power. The OA Z-scan curves could be fitted by:[8]

$$T_{\mathrm{norm}} = \frac{1}{\sqrt{\pi}[\beta I(z) L_{\mathrm{eff}}]} \int_{-\infty}^{+\infty} \ln[1 + \beta I(z) L_{\mathrm{eff}} \exp(-t^2)]\, dt \tag{5}$$

Where $I$(z) is the incident laser power at $z$ position, which can be expressed by $I_0/(1 + z^2/{z_0}^2)$. The $I_0$ is the peak power at focus point (zero position). and $z_0$ is the Rayleigh diffraction length (9.3 mm at 540 nm) of the laser beam calculated by $z_0 = \pi {\omega_0}^2/\lambda$, where $\lambda$ is wavelength, $\omega_0$ is the beam waist at focus point which obtained by calibrating with a 0.5 mm thick ZnO single crystal. $L_{\mathrm{eff}} = (1 - e^{-\alpha_0 L})/\alpha_0$ represents the effective thickness of the sample, where $L$ is the actual film thickness. Meanwhile, for SA, we employed a conventional SA model to describe the nonlinear response under high pump fluence, where the absorption coefficient can be expressed as:[9]

$$\alpha = \frac{\alpha_s}{\left(1 + {}^{I}/_{I_s}\right)} + \alpha_{\mathrm{u}} \tag{6}$$

Here, $\alpha_s$ is the saturable absorption component, $\alpha_u$ is unsaturable absorption component, $I_s$ is the saturable absorption intensity defined as the optical intensity when the optical absorbance is reduced to half of its original value. Hence, the normalized transmittance curve of OA Z-scan measurement can be presented as:[10]

$$T_{\mathrm{norm}} = \left(1 - \frac{\alpha_{\mathrm{s}} L}{1 + {}^{I}/_{I_s}}\right) \cdot \frac{1}{1 - \alpha_{\mathrm{s}} L} \tag{7}$$

Where the $\alpha_{\mathrm{s}} L$ is known as the modulation depth representing the upper limit of saturable absorption. It is worth noting that, at moderate light intensity ($I \ll I_{\mathrm{S}}$), Eq. 6 can be approximate as Eq. 4, where the linear absorption coefficient under low light field intensity as $\alpha_0 = \alpha_s + \alpha_{\mathrm{u}}$, and the NLA absorption coefficient $\beta$ equals to $-{}^{\alpha_s}/_{I_s}$.

Obviously, for SA, the value of NLA absorption coefficient $\beta$ is negative. Therefore, under moderate light intensity ($I \ll I_S$), Eq. 4 can also be regards as a standard NLA model applying to both RSA and SA process.

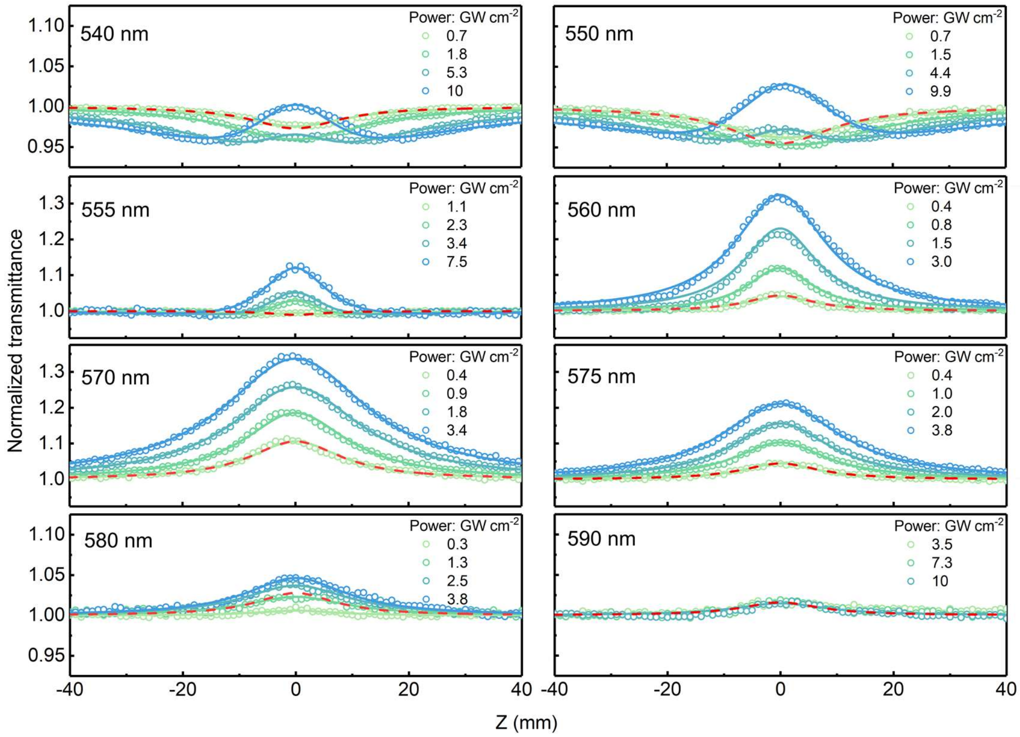


**Supplementary Figure 1. Z-scan of $(PEA)_2FAPb_2I_7$ (n=2) quasi-2D perovskite film.** OA Z-scan measurement of $(PEA)_2FAPb_2I_7$ quasi-2D perovskite film at 540 nm ~ 590 nm under different excitation intensity. The peak power at focus (0 position) of each curve is shown in top right corner. Hollow circles represent the experimental data. Solid lines are the theoretical fitting curves. Here, two different models are adapted. For 540 nm ~ 555 nm, the Z-scan results suggest apparent RSA-SA switching. Therefore, during this regime, the experimental data is fitted according to the abnormal nonlinear conversion model as Eq. 2 in main text. At wavelength above 560 nm, the RSA part is so weak that can be neglected. Hence, we adapt a standard saturable absorption model (Eq. 6). Additionally, we also adapt a standard nonlinear absorption model (Eq. 4) to extract the NLA coefficient at different wavelength under moderated pump fluence. The red dash lines denote the fitting curves. The details can be found in **Supplementary Note 2**. The calculated parameters including nonlinear absorption coefficient, saturable intensity and modulation depth are exhibited in **Supplementary Table 2**.

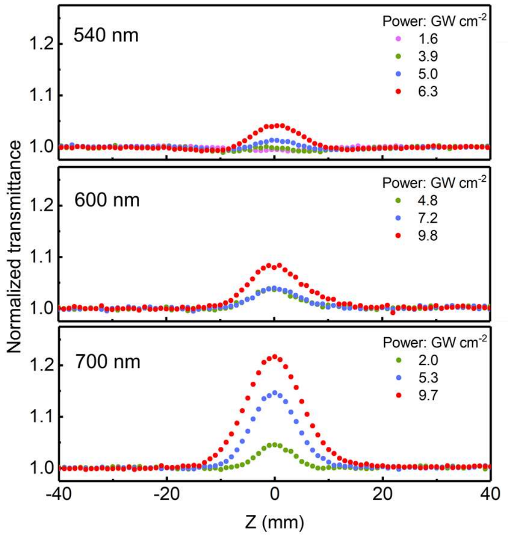


**Supplementary Figure 2. Z-scan of $FA_{0.9}MA_{0.1}PbI_3$ 3D perovskite film.** OA Z-scan results of 3D perovskite film under different excitation intensity at 540 nm, 600 nm and 700nm.

**Supplementary Table 2. Summary of the NLO parameters of $(PEA)_2FAPb_2I_7$ quasi-2D perovskite film** including NLA coefficient, saturation intensity and modulation depth using standard NLA and SA model.

| Wavelength (nm) | NLA Coefficient (cm $MW^{-1}$) | SA Intensity (GW $cm^{-2}$) | Modulation Depth |
|---|---|---|---|
| 540 | 12.75 | - | - |
| 550 | 21.80 | - | - |
| 555 | 0.75 | - | - |
| 560 | -38.20 | 3.52 | 0.546 |
| 570 | -74.80 | 1.289 | 0.401 |
| 575 | -45.50 | 1.427 | 0.2631 |
| 580 | -7.50 | 6.288 | 0.121 |
| 590 | -0.95 | 20.34 | 0.055 |

**Supplementary Note 3: Initial photon-injecting carrier density calculation.**

The femtosecond laser pulse creating carrier density is calculated by:[11]

$$n = \frac{P \cdot (1 - R - T)}{\hbar\omega \cdot S \cdot L} \tag{8}$$

Where P is the incident laser pulse energy; R and T are the reflectance and transmittance of the sample for at the excitation wavelength, 1-R-T can be obtained from the absorption spectrum, approximately; $\hbar\omega$ is the energy of the incident photon; S and L is the pump laser beam area on the sample surface and the thickness of the sample.

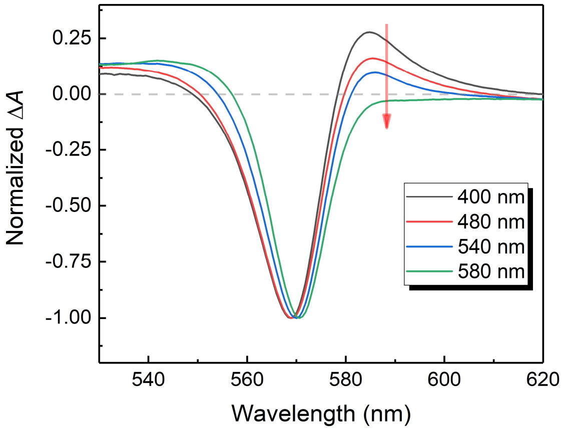


**Supplementary Figure 3. Wavelength-dependent TA spectra.** Normalized TA spectra under different pump wavelength at 200 fs delay with the injecting carrier quantity kept to be approximate as $1\times10^{19}$ $cm^{-3}$. Red arrow indicates the changing of PIA below bandgap.

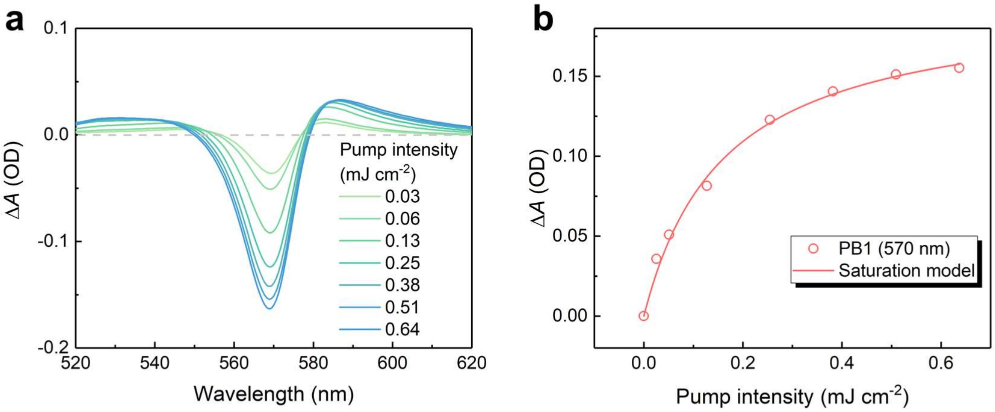


**Supplementary Figure 4. Power-dependent TA spectra.** (a) TA spectra at 200 fs delay under different pump fluence. The pump photon energy is 3.1 eV (400 nm). (b) $\Delta A$ intensity of $PIB_1$ peak as a function of pump intensity (hollow dots) fitted by standard saturation model (solid line) of $\Delta A = \frac{B\cdot I}{1+{}^{I}/_{I_S}}$, where $B$ is constant, $I_S$ is saturable intensity. The obtained $I_S$ value is 0.14 mJ $cm^{-2}$ (1.43 GW $cm^{-2}$) which is perfectly in line with that of Z-scan at 570 nm ($I_S$=1.29 GW $cm^{-2}$).

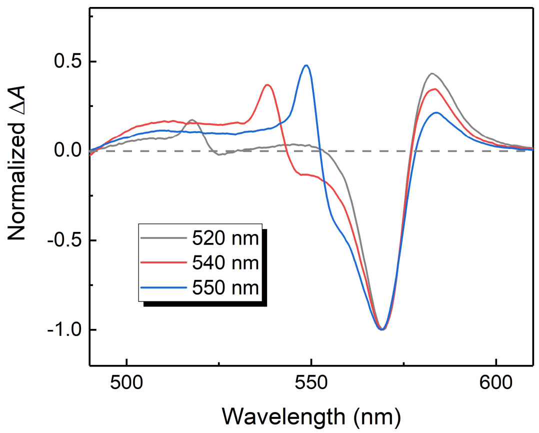


**Supplementary Figure 5. Wavelength-dependent absorption singularity.** Normalized TA spectra of quasi-2D perovskite film at 100 fs delay pumped by 520 nm, 540 nm and 550nm femtosecond laser, the pump fluence was kept at 0.13 mJ $cm^{-2}$.

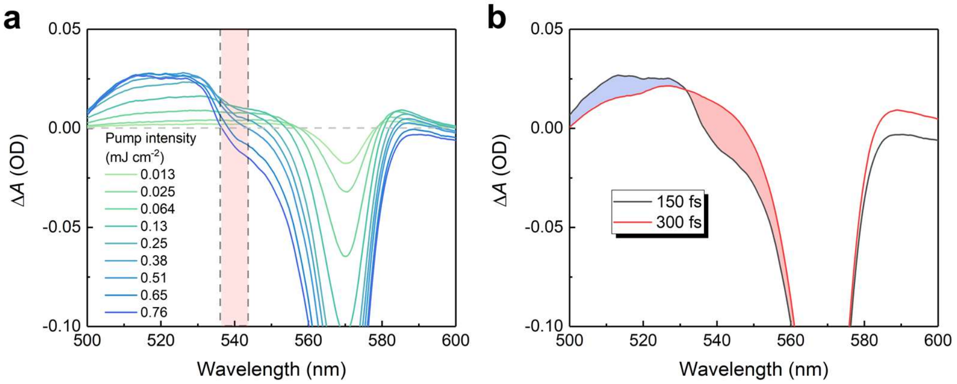


**Supplementary Figure 6. Power-dependent absorption singularity.** (a) Power-dependent TA spectra of quasi-2D perovskite film at 150 fs delay pumped by 540 nm femtosecond laser. Red region highlights $\Delta A$ changing around 540 nm. (b) The TA spectra during thermalization (150 fs) and after thermalization (300 fs). The pump intensity is 0.76 mJ $cm^{-2}$. Here, the non-thermalized carrier induced extra ΔA is labeled with blue (PIA) and red (PIB) region.

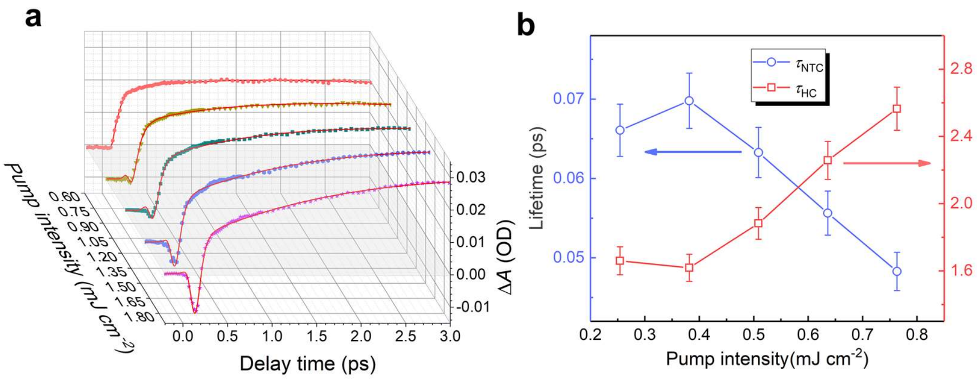


**Supplementary Figure 7. Ultrafast thermalization and hot carrier cooling kinetics.** (a) Power-dependent kinetics (circle points) around 540 nm is fitted by a three-exponential model (solid red lines) convolved with Gaussian response function. The extracted relaxation lifetime of non-thermalized carrier thermalization ($\tau_{NTC}$) and subsequent hot carrier cooling ($\tau_{HC}$) process are illustrated in (b). Error bars represent the fitting uncertainties.

**Supplementary Table 3. Summary of the fitting parameters using NLA conversion model.**

| Parameter | Quasi-2D perovskite | 3D perovskite |
|---|---|---|
| $\beta$ (cm GW$^{-1}$) | 3365.1 | 1150.4 |
| $a_0$ (cm$^{-1}$) | 43637.5 | 85660.0 |
| $L$ (cm) | $8\times10^{-6}$ | $5\times10^{-6}$ |
| $I_{s1}$ (GW cm$^{-2}$) | 2.06 | 1.85 |
| $I_{s2}$ (GW cm$^{-2}$) | 40.64 | 138.8 |

**Supplementary Note 4: Linear and transient absorption spectrum of semiconductor.**

The steady state linear absorption coefficient of a direct-bandgap 3D bulk semiconductor considering excitonic effect is given by:[11,12]

$$\alpha_{3D} = \alpha_0^{3D}\frac{\hbar\omega}{E_b}\left[\sum_{n=1}^{\infty}\frac{4\pi}{n^3}\delta\left(\Delta+\frac{1}{n^2}\right)+\Theta(\Delta)\frac{\pi e^{\frac{\pi}{\sqrt{\Delta}}}}{\mathrm{Sinh}\left(\frac{\pi}{\sqrt{\Delta}}\right)}\right] \tag{9}$$

$$\Delta = \left(\hbar\omega - E_g\right)/E_b$$

Which is known as Elliot model, where $\alpha_0^{3D}$ is a constant related to the transition matrix element, $E_b$ is the exciton binding energy, $E_g$ is the bandgap, $\hbar\omega$ represents incident photon energy, $\Theta(\Delta)$ is the unit step function, $\delta$ denotes a delta function. The first term in square bracket describes the absorption of discrete excitonic state absorption below the bandgap, while the second term describe the absorption of red side continuum states. To account for the inhomogeneous broadening, we convolve Eq. 9 by a Gaussian

function (full-width at half-maximum (FWHM) of 82 meV) to model the actual absorption spectrum. As shown in Supplementary Figure 8a, the theoretical 3D Elliot model consists well with the experimental absorption spectrum of 3D perovskite film. The contribution form exciton and continuum states absorption are labeled by green and red line, respectively. The obtained bandgap and exciton binding energy is 1.617 eV and 12.5 meV, which is in line with previous reported result.[11,13]

When the dimension of semiconductor material reduces to 2D regime, according to 2D hydrogen model, the theoretical absorption coefficient can be modified to:[12]

$$\alpha_{2\mathrm{D}} = \alpha_0^{2\mathrm{D}} \frac{\hbar\omega}{E_\mathrm{b}} \left[ \sum_{n=0}^{\infty} \frac{4}{\left(n+\frac{1}{2}\right)^3} \delta\left( \Delta + \frac{1}{\left(n+\frac{1}{2}\right)^2} \right) + \Theta(\Delta) \frac{\pi e^{\frac{\pi}{\sqrt{\Delta}}}}{\mathrm{Cosh}\left(\frac{\pi}{\sqrt{\Delta}}\right)} \right] \quad (10)$$

The confinement of the carrier enhanced the exciton absorption intensity. However, we note that in the case of hybrid layered perovskites, dielectric confinement effects cause deviations from hydrogenic behaviour.[14] Here, we only can distinguish the 1S absorption peak located at 2.17 eV under room temperature. The higher order excitonic absorption has become sightless. Here, the 1S absorption peak is modeled by an asymmetric gaussian profile given as:

$$\alpha_{1\mathrm{S}} = A \cdot \begin{Bmatrix} e^{-2\left(\frac{\hbar\omega - E_{1\mathrm{S}}}{\Gamma_1}\right)^2, \hbar\omega < E_{1\mathrm{S}}} \\ e^{-2\left(\frac{\hbar\omega - E_{1\mathrm{S}}}{\Gamma_2}\right)^2, \hbar\omega > E_{1\mathrm{S}}} \end{Bmatrix} \quad (11)$$

Where $\Gamma_1$ and $\Gamma_2$ are 43 meV and 52 meV. Whereas, even we deduce the 1S exciton absorption, there remains considerable absorption extending below 2.2 eV. This part is ascribed to continuum states absorption which is phenomenologically fitted by a assemble of Gaussian function combined with a Logistic function of:

$$\alpha = \frac{A}{1 + e^{-k(\hbar\omega - E_\mathrm{g})}} \quad (12)$$

Where *k* describes the broadening. The fitting result is shown in Supplementary Figure 8b.

For 3D perovskite with weak exciton absorption contribution, the carrier temperature can be obtained more conveniently by fitting the higher energy tails by a Maxwell–Boltzmann distribution.[11,13] However, in low-dimensional system, the confinement leads to remarkable exciton absorption peak. The bleaching signal due to exciton quenching in TA spectrum become untrivial. Fitting the TA profile according to Maxwell–Boltzmann distribution will create unacceptable deviation. Hence, in this work, based on the linear absorption fitting results, we reproduced the TA spectrum considering multiple factors[15]: (1) quenching, shifting and broadening of exciton absorption due to the presence of free carriers, (2) continuum band bleaching due to band filling defined by a quasi-Fermi energy and carrier temperature, and (3) continuum band shifting due to bandgap renormalization (BGR). The transient absorption

difference ΔA can be expressed by exciton, $\Delta A_{ex}$, adding continuous band, $\Delta A_{fc}$ modification as

$$\Delta A(\hbar\omega) = \Delta A_{\mathrm{fc}}(\hbar\omega) + \Delta A_{\mathrm{ex}}(\hbar\omega) \quad (13)$$

The continuum band contribution can be calculated by:

$$\Delta A_C(\hbar\omega) = A_C(\hbar\omega;\ E_{\mathrm{g}} - \Delta E_{\mathrm{g}})[1 - f(\hbar\omega;\ E_{\mathrm{f}};\ T)]^2 - A_{\mathrm{C}}(\hbar\omega;\ E_{\mathrm{g}}) \quad (14)$$

Where the quasi-equilibrium excited free carriers obey Fermi–Dirac distribution of

$$f(\hbar\omega;\ E_{\mathrm{f}};\ T) = \frac{1}{1 + e^{\frac{E - E_{\mathrm{f}}}{K_B T}}} \quad (15)$$

characterized by a quasi-Fermi energy level $E_f$ and a carrier temperature $T$. The exciton band contribution to the TA spectrum is given by:

$$\Delta A_{\mathrm{ex}}(\hbar\omega) = A_{\mathrm{ex}}(\hbar\omega; \Gamma;\ E_{1\mathrm{S}}) \times f_{\mathrm{b}}(n) - A_{\mathrm{ex}}(\hbar\omega; \Gamma_0;\ E_{1\mathrm{S}0}) \quad (16)$$

where $f_b(n)$ is the factor describing the reduction of exciton oscillator strength, Γ and $E_{1S}$ are the exciton bandwidth and central energy after optical excitation, $\Gamma_0$ and $E_{1\mathrm{S}0}$ are the exciton bandwidth and central energy before optical excitation, Herein, we only consider the contribution of 1S exciton state and $A_{\mathrm{ex}}(\hbar\omega; \Gamma_0;\ E_{1\mathrm{S}0})$ is the Gaussian function describing the 1S exciton absorption. As shown in Supplementary Figure 9a, our model produced the TA spectra of the 3D perovskite film within 1.6 ps successfully. The above band edge broad band PIA is attributed to BGR induced band gap shrinking and the PIB signal is dominated by the band filling of free carrier, from which we can observe the obvious hot carrier cooling process. However, for 2D perovskite, the BGR is not the main root of the band-edge PIA signal, as we discuss in main text. Here, we phenomenologically treated many-body effect induced absorption enhancement as an isolate constant term adding to Eq. 12 by $\Delta A_{\mathrm{MBE}} \cdot \theta(\hbar\omega - E_{1\mathrm{S}})$ convolving with the same Gaussian function due to inhomogeneous broadening, where $\Delta A_{\mathrm{MBE}}$ denote the PIA intensity, $\theta(\hbar\omega - E_{1\mathrm{S}})$ is a unit step function. As shown in Supplementary Fig. 9b, the calculated results coincide well with the TA spectra of quasi-2D perovskite film within 2.1 ps. The extracted fermi energy and carrier temperature is exhibited in Supplementary Fig. 9c and d.

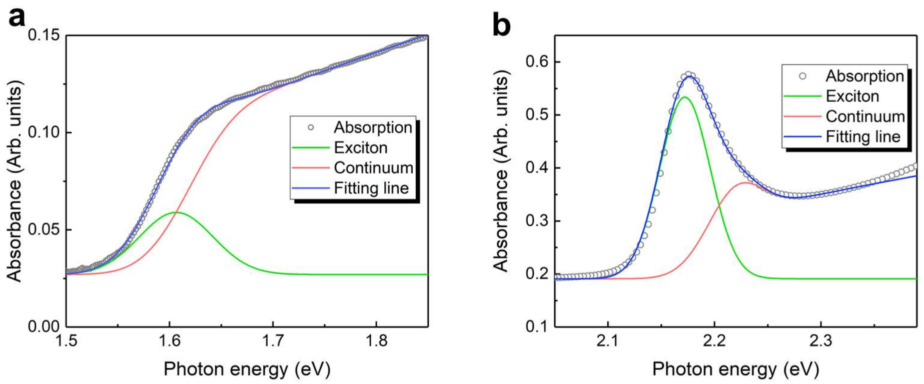

**Supplementary Figure 8. Linear absorption spectra fitting.** Linear absorption spectra of 3D and quasi-2D perovskite films fitted according to **Supplementary Note 4.** Hollow circles represent experimental data, solid lines are the fitting results.

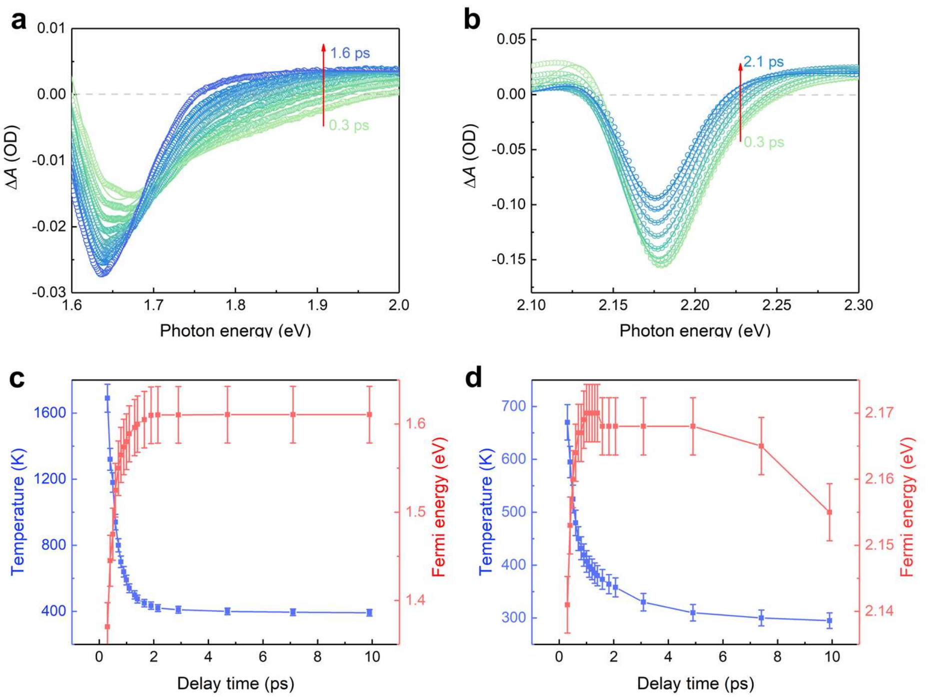


**Supplementary Figure 9. TA spectra fitting.** (a, b) TA spectra of 3D and quasi-2D perovskite film fitted according to **Supplementary Note 4.** Hollow circles represent experimental data, solid lines are the fitting results. (c, d) The extracted hot carrier temperature and Fermi energy at different delay time. Error bars represent the uncertainties in the fitting of carrier temperature and Fermi energy.

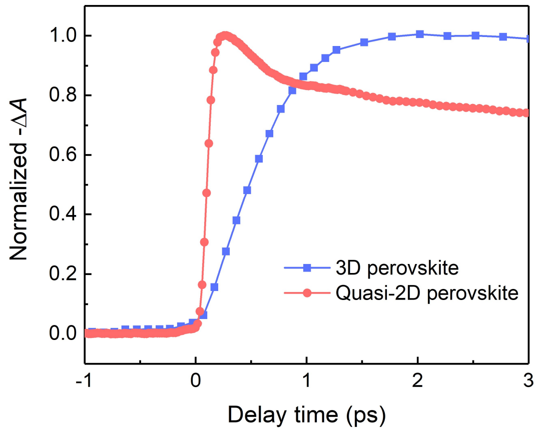

**Supplementary Figure 10. TA kinetics at band-edge.** Band-edge PIB building up kinetics curves within the first 3 ps of quasi-2D perovskite (570 nm) and 3D bulk perovskite (750 nm) film, respectively.

**Supplementary Note 5: NLA simulation on the basic of non-thermalized and hot carrier model.**

Under high excitation, large amount temporal populations of created carriers will remain in their original position of energy band which is known as non-thermalized carriers.[16] This fraction of nonequilibrium carriers will block further absorption of incoming photons at the same energy and give rise to a bleaching effect. Normally speaking, the non-thermalized carriers will undergo an ultrafast (within 100 fs) carrier-carrier scattering to form a quasi-equilibrium Fermi–Dirac distribution. This rapid intra-band relaxation process is known as thermalization. However, if the incident pump photon energy is close to bandgap, non-equilibrium carrier will not totally leave their original energy position. According to Fermi–Dirac distribution, quite amount hot carriers will remain at the excitation energy and make a non-ignorable contribution for bleaching effect. Therefore, in this work, the non-thermalized carrier and quasi-equilibrium hot carrier are simplified as two isolated energy states. Given the similar effective mass of electron and hole of perovskite, we only demonstrated the relaxation dynamic of electron in conduction band, the hole in valence band is roughly the same. For a Gaussian temporal pump pulse with uniform spatial component, the laser intensity can be denoted as:

$$I(t) = I_0 \times \frac{1}{\sigma\sqrt{2\pi}} e^{-\left(\frac{t}{\sqrt{2}\sigma}\right)^2} \tag{17}$$

The dynamic for conduction band non-thermalized electron occupation probability $f_{\mathrm{NTC}}$ can then be described by:

$$\frac{\partial f_{\mathrm{NTC}}(\hbar\omega)}{\partial t} = \alpha(I(t), \hbar\omega)\frac{1}{\mathrm{Dos}(\varepsilon)\delta}\frac{I(t)}{\hbar\omega} - \frac{f_{\mathrm{NTC}}(\hbar\omega)}{\tau_{\mathrm{NTC}}} \tag{18}$$

Where the first term represents the incident laser excited carrier, $\mathrm{Dos}(\varepsilon)$ is the density of states, $\delta$ is the incident laser FWHM in energy domain, $\alpha(I(t), \hbar\omega)$ denotes the absorbance of materials; The second term describes the ultrafast non-thermalized carrier thermalization process, $\tau_{\mathrm{NTC}}$ is the lifetime of non-thermalized carriers. Herein, the absorption of the incident photon can be expressed as Eq. 4 given in main text. After thermalization, non-thermalized carriers will decay to be quasi-equilibrium hot carriers obeying Fermi–Dirac distribution $f_{\mathrm{HC}}(\varepsilon) = 1/\left[1 + \exp\left(\frac{\varepsilon - E_{\mathrm{f}}}{K_{\mathrm{B}}T_{\mathrm{C}}}\right)\right]$. Hence, the hot carrier quantity $N_{\mathrm{HC}}$ can be given by:

$$\frac{\partial N_{\mathrm{HC}}}{\partial t} = \frac{f_{\mathrm{NTC}}(\hbar\omega) \times \mathrm{Dos}(\varepsilon)\delta}{\tau_{\mathrm{NTC}}} \tag{19}$$

$$N_{\mathrm{HC}} = \int_{E_{\mathrm{g}}}^{\infty} \mathrm{Dos}(\varepsilon) \times f_{\mathrm{HC}}(\varepsilon) d\varepsilon$$

For 2D quantum-well structure with strong quantum confinement, the density of states around band-edge can be approximatively regarded as constant according to 2D Hydrogen model. Therefore, the $\mathrm{Dos}(\varepsilon)$ in Eq. 19 can be replaced by a constant Dos. Subsequently, the equation can be resolved to give the Fermi level of hot carrier as:

$$E_{\mathrm{f}} = K_{\mathrm{b}} T \times \mathrm{Log}\left( e^{\frac{(N_{\mathrm{HC}}/\mathrm{Dos}) + E_{\mathrm{g}}}{K_{\mathrm{B}} T_{\mathrm{C}}}} - e^{\frac{E_{\mathrm{g}}}{K_{\mathrm{B}} T_{\mathrm{C}}}} \right) \tag{20}$$

If plug the calculated $E_{\mathrm{f}}$ into Fermi–Dirac function, we will obtain the occupation possibility of hot carrier, $f_{\mathrm{HC}}(\varepsilon)$. Then, Eq. 18 ~ 20 and obtained $f_{\mathrm{HC}}(\varepsilon)$ can be solved by numerical method to give the value of $f_{\mathrm{NTC}}$ and $\alpha(I(t), \hbar\omega)$. The absorbed energy per pulse can be given by:

$$W = \int_{-\infty}^{+\infty} \alpha(I(t), \hbar\omega)\, I(t) dt \tag{21}$$

The value of the parameters using in theoretical simulation can be found in Supplementary Table 4.

**Supplementary Table 4. The parameters using in NLA simulation.**

| Parameter | Description | Value |
|---|---|---|
| $\tau_{\mathrm{NTC}}$ (fs) | Non-thermalized carrier thermalization time | 50 |
| T (K) | Hot carrier temperature | 350 |
| Dos | Density of states | $2.7\times10^{23}$ |
| $\delta$ (meV) | Incident laser FWHM in energy domain | 34 |
| R (cm) | Laser beam radius on sample surface | $4\times10^{-4}$ |
| L (cm) | The thickness of the film | $8\times10^{-6}$ |

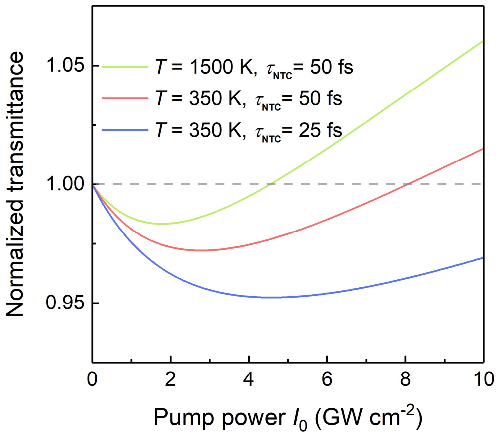

**Supplementary Figure 11. NLA simulation with different parameters.** The power-dependent transmittance evolution at 540 nm with various hot carrier temperature and thermalization time.

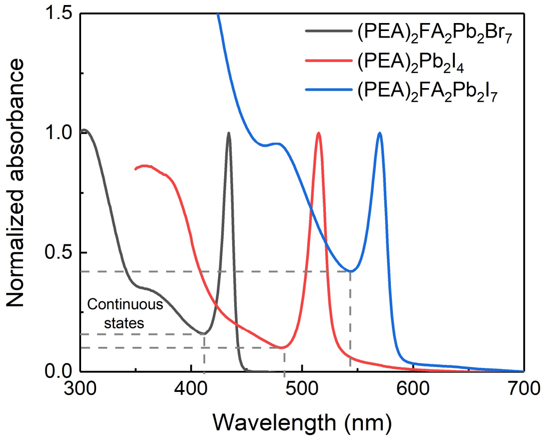


**Supplementary Figure 12. Linear absorption spectra of different 2D perovskite materials**. Absorption spectra of $(PEA)_2FAPb_2Br_7$ (n=2), $(PEA)_2PbI_4$ (n=1) and $(PEA)_2FAPb_2I_7$ (n=2) perovskite film normalized at the exciton peak. The absorption form doping states and scattering have been deducted. Dash lines denote the corresponding continuous state absorption shoulder adjacent to the excitonic absorption peak.

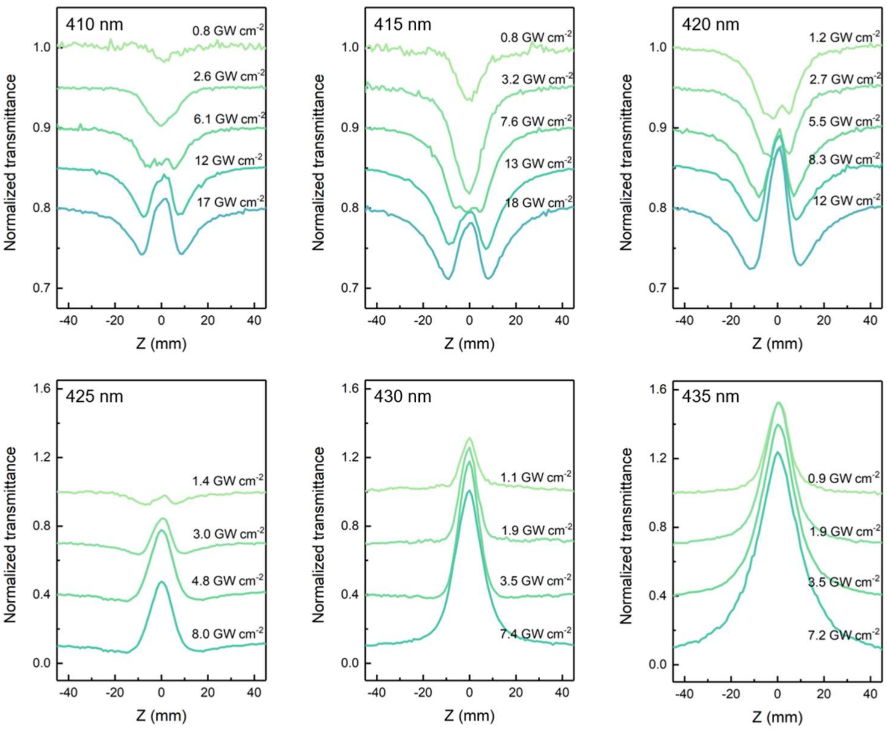


**Supplementary Figure 13. Z-scan of $(PEA)_2FAPb_2Br_7$ (n=2) quasi-2D perovskite film.** Z-scan profile evolutions of $(PEA)_2FAPb_2Br_7$ (n=2) quasi-2D perovskite film. At the blue side (410 nm~420 nm) of 1S exciton peak (435 nm), the sample demonstrates a conversion from RSA to SA with excitation intensity rising.

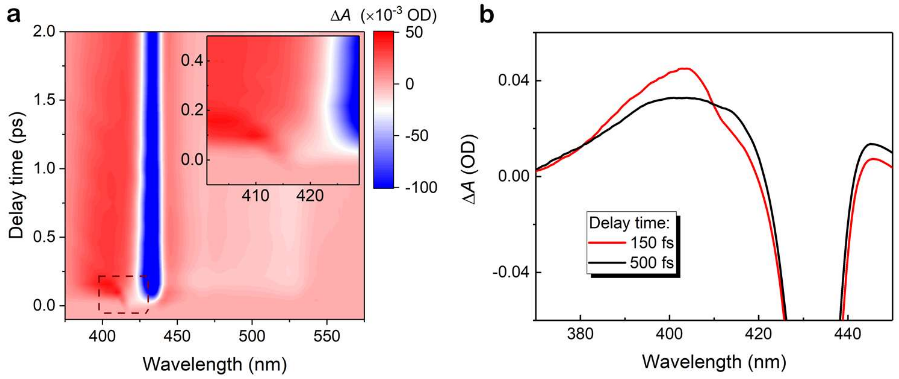


**Supplementary Figure 14. TA spectrum of $(PEA)_2FAPb_2Br_7$ (n=2) quasi-2D perovskite film.** (a) Pseudocolour representation of TA spectrum of $(PEA)_2FAPb_2Br_7$ (n=2) quasi-2D perovskite film pumped by 410 nm femtosecond laser with an excitation intensity of 0.45 mJ $cm^{-2}$. Inset: enlarged TA spectrum of the part labeled by the red frame. (b) TA spectra during (150fs) and after (500 fs) thermalization.

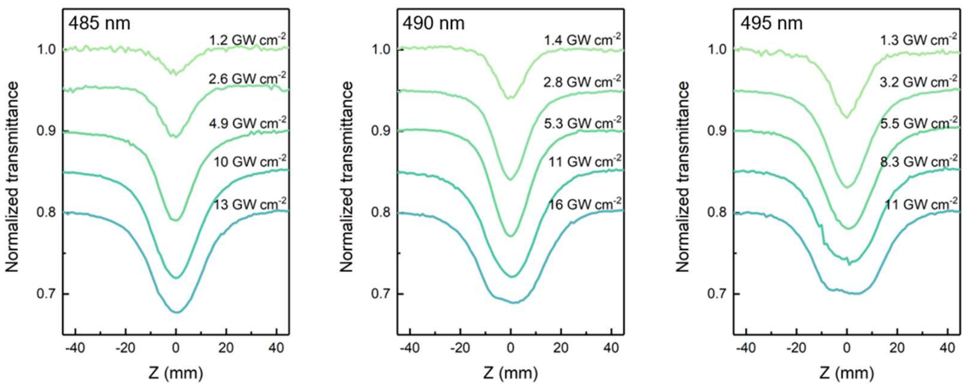


**Supplementary Figure 15. Z-scan of $(PEA)_2PbI_4$ (n=1) 2D perovskite film.** Z-scan profile evolutions of $(PEA)_2PbI_4$ (n=1) 2D perovskite film at the blue side (485 nm~495 nm) of 1S exciton peak (515 nm).

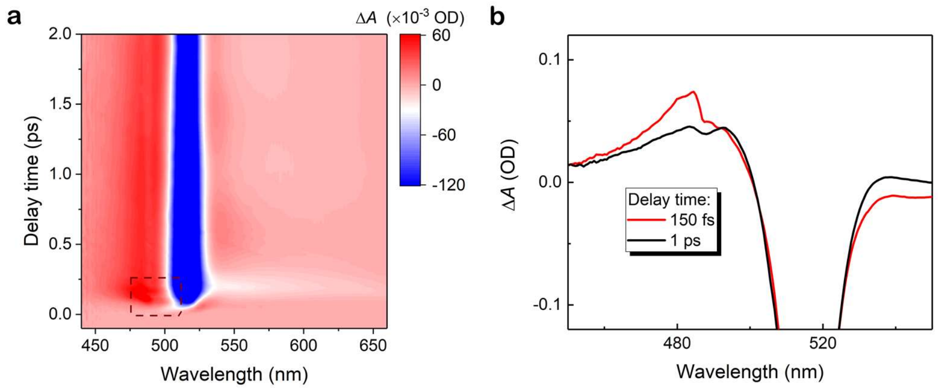


**Supplementary Figure 16. TA spectrum of $(PEA)_2PbI_4$ (n=1) 2D perovskite film.** (a) Pseudocolour representation of TA spectrum of $(PEA)_2PbI_4$ (n=1) 2D perovskite film pumped by 490 nm femtosecond laser with an excitation intensity of 0.38 mJ $cm^{-2}$. Inset: enlarged TA spectrum of the part labeled by the red frame. (b) TA spectra during (150fs) and after (1 ps) thermalization.

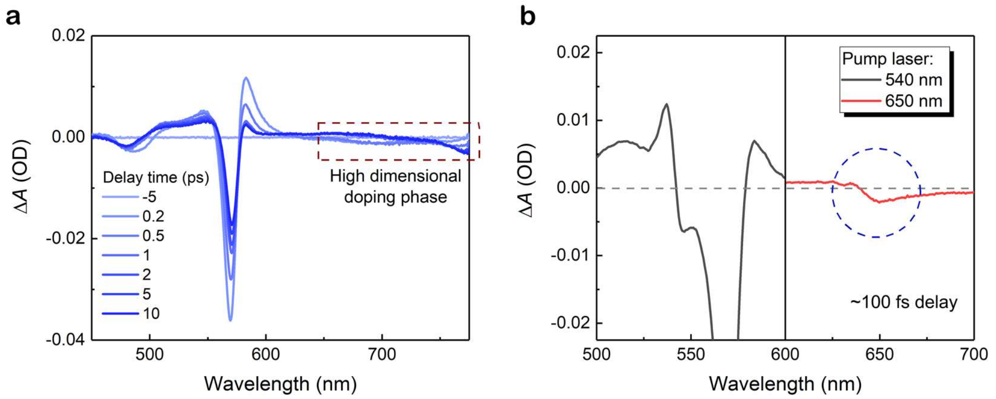


**Supplementary Figure 17. TA spectrum of the high-dimensional doping phase.** (a) Broad band TA spectra of the $(PEA)_2FAPb_2I_7$ quasi-2D perovskite film. The pump laser is 400 nm with intensity of 30 μJ cm$^{-2}$. Red frame highlights the weak signal from high dimensional doping phase indicating a low content. (b) TA spectra collected under 540 nm and 650 nm laser pumping at 100 fs delay. The pump intensity is maintained at ~0.4 mJ cm$^{-2}$. The photon energy of 650 nm is lower than the bandgap of bilayer quasi-2D perovskite phase. Hence, only the high-dimensional doping phase is excited. Blue circle denotes the nonlinear response of the high dimensional doping phase, which only result in a very weak bleaching with intensity around 0.2%.

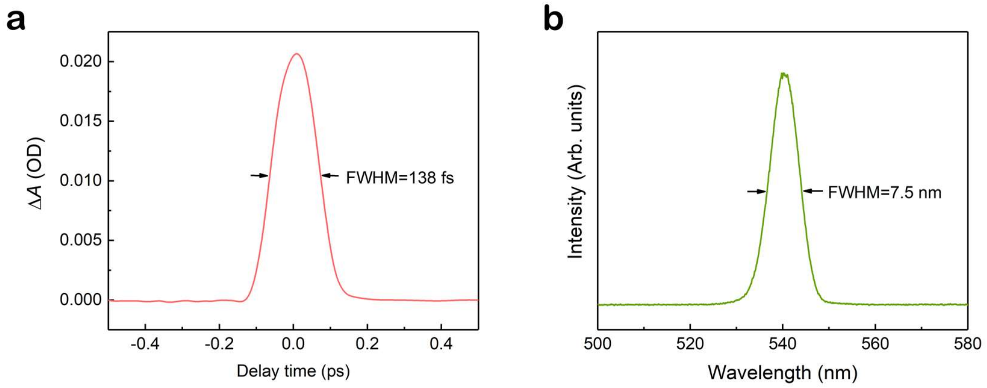


**Supplementary Figure 18.** Autocorrelation signal (a) and spectrum (b) of the used femtosecond laser pulse in experiment.

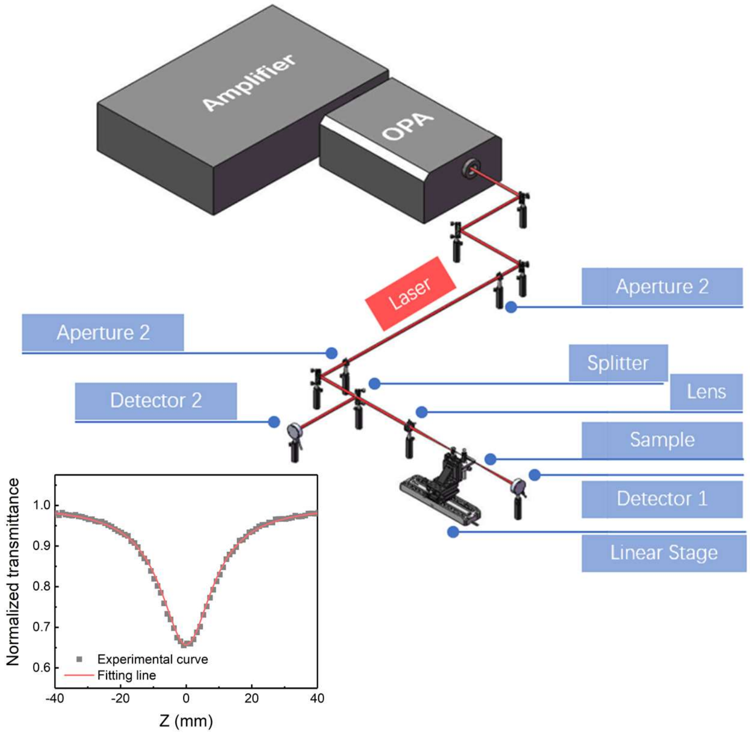


**Supplementary Figure 19. The schematic diagram of the Z-scan system.** The femtosecond laser generated from OPA is focused by a lens. The sample is fixed on a one-dimensional moving stage and scans along the laser propagation direction which is known as Z-axis. The incident laser is splitted into two beams with energy ratio of 3:7. The beam with higher power is focused on the sample and received by detector 1, which reflets the transmittance property of the sample. Another laser beam is collected by detector 2 to monitor the fluctuation of the incident laser intensity. The inset at left bottom illustrates a standard Z-scan curve of a 0.5 mm thick ZnO single crystal at 540 nm, which is fitted well with a standard nonlinear absorption model. The obtain nonlinear absorption coefficient is 7.2 cm $GW^{-1}$ which is coincident with previous reported result (5 cm $GW^{-1}$).[17]

## References


1 Liu, J. *et al.* Nonlinear optical absorption properties of InP nanowires and applications as a saturable absorber. *Photonics Res.* **8**, 1035-1041 (2020).

2 Deng, X. *et al.* Intensity threshold in the conversion from reverse saturable absorption to saturable absorption and its application in optical limiting. *Opt. Commun.* **168**, 207-212 (1999).

3 Hughes, S., Spruce, G., Wherrett, B., Welford, K. & Lloyd, A. The saturation limit to picosecond, induced absorption in dyes. *Opt. Commun.* **100**, 113-117 (1993).

4 Hughes, S., Spruce, G., Wherrett, B. S. & Kobayashi, T. Comparison between the optical limiting behavior of chloroaluminum phthalocyanine and a cyanine dye. *J. Appl. Phys.* **81**, 5905-5912 (1997).

5 Li, C., Si, J., Yang, M., Wang, R. & Zhang, L. Excited-state nonlinear absorption in multi-energy-level molecular systems. *Phys. Rev. A* **51**, 569-575 (1995).

6 Si, J. *et al.* Nonlinear excited state absorption in cadmium texaphyrin solution. *Appl. Phys. Lett.* **64**, 3083-3085 (1994).

7 Przhonska, O. V. *et al.* Nonlinear light absorption of polymethine dyes in liquid and solid media. *JOSA B* **15**, 802-809 (1998).

8 Gu, C. *et al.* Giant and multistage nonlinear optical response in porphyrin-based surface-supported metal-organic framework nanofilms. *Nano Lett.* **19**, 9095-9101 (2019).

9 Sutherland, R. L. *Handbook of Nonlinear Optics*. (CRC press, 2003).

10 Wang, K. *et al.* Ultrafast saturable absorption of two-dimensional MoS2 nanosheets. *ACS Nano* **7**, 9260-9267 (2013).

11 Yang, Y. *et al.* Observation of a hot-phonon bottleneck in lead-iodide perovskites. *Nat. Photonics* **10**, 53-59 (2015).

12 Haug, H. & Koch, S. W. *Quantum Theory of The Optical and Electronic Properties of Semiconductors: Fivth Edition*. (World Scientific Publishing Company, 2009).

13 Fu, J. *et al.* Hot carrier cooling mechanisms in halide perovskites. *Nat. Commun.* **8**, 1300 (2017).

14 Passarelli, J. V. *et al.* Tunable exciton binding energy in 2D hybrid layered perovskites through donor–acceptor interactions within the organic layer. *Nature chemistry* **12**, 672-682 (2020).

15 Lim, J. W. M. *et al.* Hot carriers in halide perovskites: how hot truly? *J. Phys. Chem. Lett.* **11**, 2743-2750 (2020).

16 Richter, J. M. *et al.* Ultrafast carrier thermalization in lead iodide perovskite probed with two-dimensional electronic spectroscopy. *Nat. Commun.* **8**, 376 (2017).

17 Van Stryland, E. W., Woodall, M. A., Vanherzeele, H. & Soileau, M. J. Energy band-gap dependence of two-photon absorption. *Opt. Lett.* **10**, 490-492 (1985).
